# Prospective study of an adalimumab combined with partial enteral nutrition in the induction period of Crohn’s disease

**DOI:** 10.1007/s00011-023-01828-7

**Published:** 2024-01-02

**Authors:** Sisi Zhou, Zeyu Huang, Wenjing Hou, Yiting Lin, Jing Yu

**Affiliations:** Department of Gastroenterology, First Affiliated Hospital of Shantou University Medical College, No. 57 Changping Road, Shantou, 515041 China

**Keywords:** Crohn’s disease, Exclusive enteral nutrition, Partial enteral nutrition, Adalimumab

## Abstract

**Background:**

Adalimumab monotherapy can suppress gut inflammation and induce remission in active Crohn’s disease but has some limitations. Exclusive enteral nutrition (EEN) is recommended for patients with mild to moderate Crohn’s disease (CD), but implementation is challenging.

**Aim:**

To evaluate the effectiveness of adalimumab combined with partial enteral nutrition (PEN) in the induction therapy for Crohn’s disease.

**Methods:**

A prospective cohort study was designed and a total of 56 patients with active CD who met the criteria for enteral nutrition (EN) treatment in our hospital were selected. The baseline data of all patients were collected including age, sex and other general information. The changes in fecal calprotectin, C-reactive protein (CRP), albumin(Alb), hemoglobin (Hb), platelets (Plt), erythrocyte sedimentation rate (ESR), Crohn’s disease activity index score (CDAI), simple endoscopic score (SES-CD) and body mass index (BMI) were compared between the adalimumab combined with enteral nutrition (ADA+EN) group (*N* = 37) the adalimumab group (ADA) (*N* = 19) at week 0 (W0) and treatment outcomes at week 12(W12). Additionally, the differences between the two groups before and after treatment were evaluated. Then the ADA+EN group was divided into an adalimumab combined with exclusive enteral nutrition subgroup (ADA+EEN) and an adalimumab combined with partial nutrition subgroup (ADA+PEN) according to enteral nutrition intake. The changes in fecal calprotectin, CRP, Alb, Hb, Plt, ESR and CDAI, SES-CD and BMI were compared between the  ADA+EEN group and the ADA+PEN group at week 0 (W0) and treatment outcomes at week 12(W12). The differences between the two groups before and after treatment were evaluated. To evaluate the effectiveness of the two treatments on patients’ quality of life, nutritional recovery and body composition, patients in the ADA+EN group were needed to complete the Inflammatory Bowel Disease Questionnaire (IBDQ), EQ-5D-5L, the EuroQol visual analogue scale (EQ-VAS) and body composition analysis.A total of 28 patients completed all questionnaires and body composition analyses at week 0 and week 12, including 10 patients in the ADA+EEN group and 18 patients in the ADA+PEN group, respectively. The differences of in IBDQ, EQ-5D-5L and body composition analysis were compared between the two groups at week 0 (W0) and treatment outcomes at week 12(W12). Additionally, the differences between the two groups before and after treatment were evaluated.

**Results:**

These investigated indexes such as calprotectin, Hb, Plt, ESR, Alb, BMI, CRP, CDAI and SES-CD scores were significantly different before and after treatment  in the ADA+EN group (*p* < 0.01). However, fecal calprotectin, Hb, SES-CD scores and Alb in the ADA group were not statistically significantly different from W0 to W12 (*p* > 0.05). The fecal calprotectin and CDAI scores in the ADA+EN group were significantly lower than those in the ADA group after treatment. The differences in all factors before and after treatment between the ADA+PEN group and the ADA+EEN group were statistically significant (*p* < 0.05). However, there was no significant difference between the two groups at week 12 (*p* > 0.05).

**Conclusion:**

Adalimumab combined with EN are more effective than ADA monotherapy in terms of endoscopy and clinical remission. By comparing the investigated indicators such as calprotectin, Hb, Plt, ESR ,CRP and SES-CD scores, it was proven that adalimumab combined with partial enteral nutrition or exclusive enteral nutrition has the same remission effect in induced Crohn’s disease. The combination of biological agents and partial nutrition can improve medical order compliance, psychological burden and quality of life. Therefore, adalimumab combined with partial nutrition can be used as the first-line treatment for CD induced remission.

## Introduction

Crohn’s disease (CD) is a chronic inflammatory bowel disease (IBD) of the gastrointestinal tract and also a progressive disease that leads to bowel damage and disability. All segments of the gastrointestinal tract can be affected, the most common segments include being the terminal ileum and colon. Inflammation is typically segmental, asymmetrical, and transmural. Most patients present with an inflammatory phenotype at the time of diagnosis but over time complications (strictures, fistulas, or abscesses) will develop in half of patients, often resulting in surgery. Endoscopy remains the gold standard for diagnosis [1]. Fecal calprotectin is a helpful test that should be employed to help differentiate inflammatory bowel disease (IBD) from irritable bowel syndrome (IBS) [2]. At present, there are different types of drugs for the treatment of IBD, and the effective utilization rate of drugs for this disease has become the focus of research in recent years. Anti-Tnf-α drugs are one of the important for the treatment of IBD.Adalimumab is a recombinant fully human immunoglobulin (IgG1) monoclonal antibody containing a human polypeptide sequence. Adalimumab has a high affinity for soluble TNF-α and can effectively counteract the biological functions of TNF-α by blocking the interaction between TNF-α and its receptors p55 and p75 [3]. The CHARM（Crohn’s trial of the fully human antibody adalimumab for remission maintenance) research showed that the remission rate in the ADA group was 40% at week 26 and 36% at week 56 [4].The EXTEND(extending the safety and efficacy of adalimumab through endoscopic healing) research showed that 27% of patients achieved mucosal healing and 52% of patients achieved endoscopic remission in the treatment group at week 12 [5].

Therefore, it is important to find a safe and effective way to improve the success rates of induction and remission. Literature review suggested that biological agents combined with EEN could effectively promote the remission in patients with active stages of Crohn’s disease. In a prospective study, 41 patients with active CD complicated with fistulas, abdominal abscess or strictures were treated with EEN for 12 weeks, and after which CDAI was significantly reduced. The complete remission rate was 80.5% and mucosal healing was observed in 47% of the patients [6]. A meta-analysis of eight RCTs  researches involving 226 children with CD was conducted to compare the efficacy of total enteral nutrition and hormone therapy. The results showed that the rate of CD remission induced by EEN was similar to the rate of CD remission induced by hormone drugs [7]. Therefore, is the use of a biologic agent combined with enteral nutrition is more effective than the use of a biologic agent alone during the induction of remission in patients with CD?

The most important barriers to its use are the repetitive and poor taste of enteral formulas and the heavy dietary restriction EEN places on patients, as they are not allowed to consume any other food over a long period of time. This is also the main reason why EEN therapy is not well accepted or adhered to by many patients [8]. This has led to research interest in the effectiveness of partial enteral nutrition (PEN) combined with an unrestricted oral diet. Wilschanski et al. conducted a retrospective study to identify the effect of nocturnal supplementary enteral nutrition delivered in combination with a normal diet on the duration of remission in pediatric CD. Forty-seven patients (72%) achieved remission, 28 of whom continued on supplementary feeds maintaining remission for a significantly increased duration compared with those who resumed an otherwise normal diet [9]. In the CERISIER trial, which was conducted in Japan by Hisamatsu et al. the combination of PEN and dose escalation of anti-Tnf-α agents was superior to dose escalation alone in patients with secondary loss of response [10]. A study of pediatric patients with CD suggests that PEN combined with medical therapy such as corticosteroids, azathioprine, anti-TNF drugs, or methotrexate that has the potential to prolong remission [11]. Most of the above studies were based on data from children, and there are limited data from clinical studies in adults. Therefore, we wondered whether the introduction of PEN intervention in addition to Anti-Tnf-α drugs could achieve the same remission induction rate in CD as the combination therapy with EEN.

## Methods

From November 2020 to March 2023, all patients with active CD (newly diagnosed and those with exacerbations) treated in a single tertiary center, who fulfilled the inclusion criteria for EN treatment, were prospectively included in the study. Our objectives for this study were to assess the efficacy and tolerability of a novel dietary intervention for CD, based on an unlimited oral diet and PEN, and to compare it with the “gold standard” but difficult to implement dietary intervention of EEN. Patients (aged 14–75 years) defined by enrolled. The inclusion criteria was clinically and endoscopically active CD, defined as a Crohn’s disease activity index score (CDAI) ≥ 150 and that had evidence for active inflammation at enrollment, such as elevated C-reactive protein (CRP) > 5 g/L and erythrocyte sedimentation rate (ESR) > 20 mm/h or calprotectin > 50 μg/g. Patients were allowed to use biologics in the past because their effect would not affect the week 12 endpoints.Patients were allowed to use of a proton pump inhibitor if ulcers or erosion were documented in the stomach or duodenum. 

The exclusion criteria were CDAI < 150, SES-CD ≤ 3, penetrating disease (abscess or fistula), active perianal disease not draining well, fixed strictures or small bowel obstruction, changes in maintenance treatment, or having received steroids in the last 3 months prior to inclusion. Patients were also excluded if they had high fever > 38.5 °C, intercurrent or opportunistic infection or refused to use the diet. Signed informed consent was obtained from all participating families or patients before enrollment. This study received ethical approval from the hospital ethics committee which is required for prospective studies or collection of patient data for clinical reports（Ethical Approval No.B-2022-002）.

**Fig. 1:**
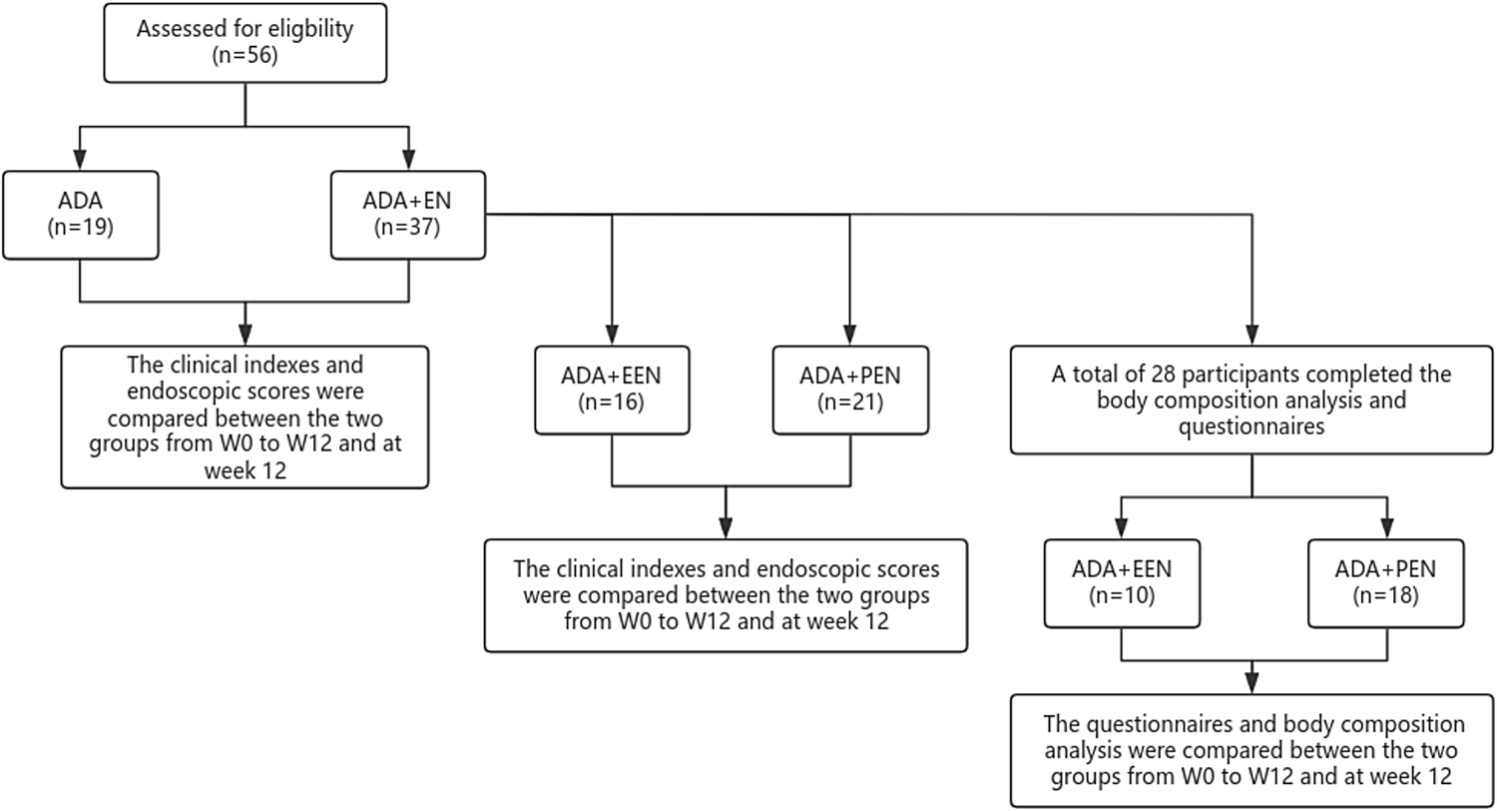
Flow chart of patients treated with the Crohn’s disease combined with enteral nutrition

In this prospective study, Fig. 1 shows that all eligible patients were simply randomly assigned to receive adalimumab combined with enteral nutrition (ADA+EN) or ADA monotherapy. Finally, a total of 37 patients in the ADA+EN group and 19 patients in ADA group were collected. Clinical laboratory indexes such as fecal calprotectin, CRP, Alb, Hb, Plt, ESR, CDAI, SES-CD and BMI were recorded and compared at week 0 (W0) and treatment outcomes week 12(W12). Additionally, the differences between the two groups before and after treatment were evaluated.

We divided 37 patients in the biological agent combined enteral nutrition group into the adalimumab combined with exclusive enteral nutrition (ADA+EEN) group (*N* = 16) and adalimumab combined with partial enteral nutrition (ADA+PEN) group (*N* = 21). Clinical laboratory indexes such as fecal calprotectin, CRP, Alb, Hb, PLT, ESR, CDAI, SES-CD and BMI were recorded and compared at week 0 (W0) and treatment outcomes week 12(W12). However, only 28 participants in the ADA+EN group completed the IBDQ, EQ-5D-5L, EQ-VAS and body composition analysis. Changes in quality of life were compared between the two groups by examining responses to a questionnaire, and the nutritional recovery was observed by body composition analysis (Fig. 1).

Five items of the IBDQ were measured, including intestinal symptoms, systemic symptoms, emotional function, social function and total score. Six items of the EQ-5D-5L were assessed, including mobility, self-care, daily activities, pain or discomfort, anxiety or depression and physical condition. A body composition analyzer was also used to measure body fat mass, body muscle mass, body water, inorganic salts and basal metabolism.

All patients received subcutaneous injections of the adalimumab biosimilar 160 mg in the first week, 80 mg at 2 weeks, and injections of 40 mg every 2 weeks thereafter. In the ADA+EEN group which all nutrients were provided by oral feeding at least 30–35 kcal·kg^−1^·d^−1^. In the ADA+PEN group, the daily energy requirement was also calculated as 30–35 kcal·kg^−1^·d^−1^, and at least half of the energy requirement was provided by nutrition powder,the rest of the energy was provided by a normal diet. In the ADA group, all energy sipplies were provided by a normal diet. All enteral nutrition was administered orally only.

SPSS 27 software was used to analyze the data. Quantitative data such as age, BMI, CRP, fecal calprotectin, Hb, PLT, ESR, CDAI score, SES-CD score and Alb are described as the mean ± standard deviation. A *t* test was used for comparisons between different groups. Qualitative data such as gender, location, and the first use of biological agents were described by frequency and percentage, and the comparison between different groups was performed by chi-square (*X*^2^) test. *p* < 0.05 were considered statistically significant.

## Results

Fifty-six patients with CD were divided into an adalimumab combined with EN group (*N* = 37) and an adalimumab monotherapy group (*N* = 19).

The basic data of the two groups were compared, and only BMI and Hb were significantly different (*p* < 0.05).Other factors were not statistically significant. Then we divided the biological agents combined with EN group into the ADA+EEN subgroup (*N* = 16) and ADA+PEN subgroup (*N* = 21).There was no significant difference in any factors between the two subgroups (*p* > 0.05) (Table 1).

**Table 1:** The baseline characteristics of the patients were compared between the ADA group and ADA+EN group, and the baseline characteristics of the patients were compared between the ADA+EEN group and ADA+PEN group

Variable	ADA+EN (*N* = 37)	ADA (*N* = 19)	*t*	*P*	ADA+PEN (*N* = 21)	ADA+EEN (*N* = 16)	*t*	*P*
Age	30.32 ± 13.12	29.89 ± 13.15	0.12	0.9082	33.67 ± 14.67	25.94 ± 9.52	1.83	0.0755
BMI (kg/m^2^)	17.97 ± 2.41	19.26 ± 1.92	− 2.03	0.0473	17.85 ± 1.86	18.13 ± 3.05	− 0.34	0.7342
CRP (mg/L)	12.11 ± 9.43	8.78 ± 6.83	1.36	0.1791	11.66 ± 8.32	12.70 ± 10.98	− 0.33	0.7445
Fecal calprotectin (mg/kg)	290.20 ± 151.56	264.40 ± 120.30	0.64	0.5220	282.80 ± 160.00	299.90 ± 144.30	− 0.34	0.7392
Hemoglobin (g/L)	111.10 ± 23.06	124.60 ± 16.60	− 2.26	0.0276	109.50 ± 25.40	113.30 ± 20.17	− 0.48	0.6329
Platelets (g/L)	314.50 ± 93.28	282.60 ± 76.65	1.28	0.2052	317.40 ± 98.26	310.70 ± 89.34	0.21	0.8311
ESR (mm/h)	22.43 ± 12.62	23.68 ± 15.67	− 0.32	0.7476	23.05 ± 13.02	21.63 ± 12.44	0.34	0.7392
CDAI score	346.50 ± 124.00	319.90 ± 101.20	0.81	0.4241	341.40 ± 118.00	353.20 ± 135.00	− 0.28	0.7797
SES-CD score	12.14 ± 7.19	9.11 ± 7.63	1.46	0.1494	12.43 ± 7.31	11.75 ± 7.25	0.28	0.7806
Albumin (g/L)	37.33 ± 5.55	39.14 ± 5.84	− 1.13	0.2615	37.64 ± 5.53	36.94 ± 5.72	0.38	0.7077
Gender			*X*^2^ = 0.007	0.9351			*X*^2^ = 0.524	0.4690
Female, *n* (%)	16 (43.24%)	8 (42.11%)			8 (38.10%)	8 (50.00%)		
Male, *n* (%)	21 (56.76%)	11 (57.89%)			13 (61.90%)	8 (50.00%)		
Location, *n* (%)			*X*^2^ = 3.571	0.4671			*X*^2^ = 2.917	0.5718
L1, *n*(%)	2 (5.41%)	3 (15.79%)			1 (6.25%)	2 (5.41%)		
L2, *n*(%)	9 (24.32%)	3 (15.79%)			5 (31.25%)	9 (24.32%)		
L3, *n*(%)	21 (56.76%)	12 (63.16%)			9 (56.25%)	21 (56.76%)		
L3 + L4, *n*(%)	2 (5.41%)	1 (5.26%)			1 (6.25%)	2 (5.41%)		
L4, *n*(%)	3 (8.11%)	0			0	3 (8.11%)		
Use of biologics for the first time			*X*^2^ = 0.291	0.5895			*X*^2^ = 1.627	0.2022
No, *n*(%)	11 (29.73%)	7 (36.84%)			8 (38.10%)	3 (18.75%)		
Yes, *n*(%)	26 (70.27%)	12 (63.16%)			13 (61.90%)	13 (81.25%)		

*ADA+EN*: Adalimumab combined with  enteral nutrition, *ADA*: adalimumab, *ADA+EEN*: Adalimumab combined with exclusive enteral nutrition, *ADA+PEN*: Adalimumab combined with partial enteral nutrition

The ADA+EN group and ADA group were compared from W0 to W12 and we found that there were significant differences in BMI, CRP, fecal calprotectin, Hb, Plt, ESR, CDAI score, SES-CD score and Alb before and after treatment in the ADA+EN group (*p* < 0.01). There were also significant differences in BMI, CRP, Plt, ESR and CDAI scores before and after treatment in the ADA group (*p* < 0.05). However, there were no significant differences in fecal calprotectin, Hb, Alb and SES-CD scores before and after treatment (*p* > 0.05). Independent sample *t* test were used to compare the indicators between the two groups at week 12. There were significant differences in fecal calprotectin, CDAI score and Alb between the two groups (*p* < 0.05). Fecal calprotectin and CDAI scores were significantly lower in the ADA+EN group than in the ADA group after treatment. The level of albumin among patients in the ADA+EN group was higher than that among patients in the ADA group (Table 2).

**Table 2:** Comparison of the ADA+EN group and ADA group before and after treatment

Group	*n*	BMI (kg/m^2)^		*P*	CRP (mg/L)		*P*	Fecal calprotectin (mg/kg)		*P*
		Week 0	Week 12		Week 0	Week 12		Week 0	Week 12	
ADA+EN	37	17.97 ± 2.41	18.78 ± 2.68	0.0013	12.11 ± 9.43	3.47 ± 2.62	< 0.0001	290.20 ± 151.56	151.99 ± 78.82	< 0.0001
ADA	19	19.26 ± 1.92	19.94 ± 1.98	0.001	8.78 ± 6.83	4.59 ± 3.09	0.008	290.20 ± 151.56	213.75 ± 107.26	0.1745
*t*	–	− 2.03	− 1.67		1.36	− 1.42		0.64	− 2.45	
*P*	–	0.0473	0.1016		0.1791	0.1621		0.522	0.0176	

Group	*n*	Hemoglobin (g/L)		*P*	Platelets (g/L)		*P*	ESR (mm/h)		*P*
		Week 0	Week 12		Week 0	Week 12		Week 0	Week 12	
ADA+EN	37	111.10 ± 23.06	128.49 ± 19.89	< 0.0001	314.50 ± 93.28	231.41 ± 55.46	< 0.0001	22.43 ± 12.62	12.35 ± 10.80	0.0002
ADA	19	124.60 ± 16.60	124.47 ± 15.69	0.9352	282.60 ± 76.65	238.26 ± 63.06	0.0144	23.68 ± 15.67	13.32 ± 8.76	0.0042
*t*	–	− 2.26	0.76		1.28	− 0.42		− 0.32	− 0.34	
*P*	–	0.0276	0.4478		0.2052	0.6775		0.7476	0.7381	

Group	*n*	CDAI score		*P*	SES-CD score		*P*	Albumin (g/L)		*P*	DC (µg/ml)		*P*
		Week 0	Week 12		Week 0	Week 12		Week 0	Week 12		Week 0	Week 12	
ADA+EN	37	346.50 ± 124.00	149.61 ± 76.36	< 0.0001	12.14 ± 7.19	5.95 ± 5.99	< 0.0001	37.33 ± 5.55	42.14 ± 3.60	< 0.0001	–	12.02 ± 7.84	–
ADA	19	319.90 ± 101.20	208.73 ± 94.07	0.0014	9.11 ± 7.63	6.47 ± 6.00	0.1968	39.14 ± 5.84	39.56 ± 4.38	0.6783	–	10.06 ± 6.09	–
*t*	–	0.81	− 2.53		1.46	− 0.31		− 1.13	2.36		–	0.95	
*P*	–	0.4241	0.0142		0.1494	0.7566		0.2615	0.0222		–	0.3472	

*ADA+EN*: Adalimumab combined with  enteral nutrition, *ADA*: adalimumab, *DC*: drug concentration

There were significant differences in all factors such as fecal calprotectin, CRP, Alb, Hb, Plt, ESR, CDAI score, SES-CD score and BMI) between the ADA+PEN group and the ADA+EEN group from W0 to W12. (*p* < 0.05). However, there was no significant difference in the laboratory indexes, CDAI score and SES-CD score at week 12 between the two groups (*p* > 0.05) (Table 3).

**Table 3:** Comparison of the ADA+PEN group and ADA+EEN group before and after treatment

Group	*n*	BMI (kg/m^2)^		*P*	CRP (mg/L)		*P*	Fecal calprotectin (mg/kg)		*P*
		Week 0	Week 12		Week 0	Week 12		Week 0	Week 12	
ADA+EEN	16	18.13 ± 3.05	19.13 ± 3.51	0.0287	12.70 ± 10.98	2.90 ± 2.57	0.0022	299.90 ± 144.30	142.71 ± 72.81	< 0.0001
ADA+PEN	21	17.85 ± 1.86	18.51 ± 1.86	0.0224	11.66 ± 8.32	3.91 ± 2.64	0.0003	282.80 ± 160.00	159.06 ± 84.17	0.0002
*t*	–	− 0.34	− 0.63		− 0.33	1.16		− 0.34	0.62	
*P*	–	0.7342	0.5339		0.7445	0.2539		0.7392	0.5394	

Group	*n*	Hemoglobin (g/L)		*P*	Platelets (g/L)		*P*	ESR (mm/h)		*P*
		Week 0	Week 12		Week 0	Week 12		Week 0	Week 12	
ADA+EEN	16	113.30 ± 20.17	130.13 ± 18.15	0.0023	310.70 ± 89.34	212.31 ± 51.71	< 0.0001	21.63 ± 12.44	12.25 ± 12.18	0.0455
ADA+PEN	21	109.50 ± 25.40	127.24 ± 21.47	0.0003	317.40 ± 98.26	245.95 ± 54.94	0.0028	23.05 ± 13.02	12.43 ± 9.94	0.0015
*t*	–	− 0.48	− 0.43		0.21	1.89		0.34	0.05	
*P*	–	0.6329	0.6680		0.8311	0.0668		0.7392	0.9611	

Group	*n*	CDAI score		*P*	SES-CD score		*P*	Albumin (g/L)		*P*	DC (µg/ml)		*P*
		Week 0	Week 12		Week 0	Week 12		Week 0	Week 12		Week 0	Week 12	
ADA+EEN	16	353.20 ± 135.00	129.52 ± 72.74	< 0.0001	11.75 ± 7.25	4.38 ± 5.44	0.0057	36.94 ± 5.72	42.01 ± 3.61	0.0136	–	12.40 ± 5.20	–
ADA+PEN	21	341.40 ± 118.00	164.92 ± 77.18	< 0.0001	12.43 ± 7.31	7.14 ± 6.26	0.0009	37.64 ± 5.53	42.24 ± 3.68	0.0003	–	11.73 ± 9.50	–
*t*	–	− 0.28	1.42		0.28	1.41		0.38	0.19		–	− 0.27	
*P*	–	0.7797	0.1655		0.7806	0.1676		0.7077	0.8518		–	0.7856	

*ADA+EEN*: Adalimumab combined with exclusive enteral nutrition, *ADA+PEN*: Adalimumab combined with partial enteral nutrition, *DC*: drug concentration

Body composition analysis included body fat percentage, muscle, body water, inorganic salts and basal metabolic rate (BMR). Patients in the ADA+PEN group and ADA+EEN group were assessed for their physical condition via noninvasive rapid measurement from week 0 to week 12 and between the groups at week 12. In the ADA+PEN group, there were significant differences in body fat percentage, muscle, body water and BMR before and after treatment (*p* < 0.05); these parameters were significantly higher after treatment than before treatment. In the ADA+EEN group, muscle, body water and BMR were significantly different before and after treatment (*p* < 0.05); more specifically, these parameters were significantly higher after treatment than before treatment. There was no significant difference in body water, inorganic salts or BMR between the ADA+PEN group and the ADA+EEN group at week 0 and week 12 (*p* > 0.05) (Table 4).

**Table 4:** Body fat percentage, muscle mass, body water, inorganic salts, and basal metabolism rate were compared between the ADA+PEN group and ADA+EEN group at week 0 and week 12

Group	*n*	Fat percentage		*P*	Muscle		*P*	Body water		*P*
		Week 0	Week 12		Week 0	Week 12		Week 0	Week 12	
ADA+EEN	10	8.82 ± 2.92	9.73 ± 3.57	0.1449	34.52 ± 1.28	37.55 ± 3.67	0.0218	27.97 ± 5.11	29.69 ± 5.52	0.0368
ADA+PEN	18	6.01 ± 3.08	6.94 ± 3.80	0.0372	38.74 ± 5.47	40.31 ± 5.51	0.0210	29.46 ± 4.15	30.38 ± 4.37	0.0190
*t*	–	– 2.36	– 1.90	–	3.13	1.41	–	0.84	0.36	–
*P*	–	0.0263	0.0689	–	0.0053	0.1690	–	0.4105	0.7192	–

Group	*n*	Inorganic salts		*P*	Basal metabolic rate		*P*
		Week 0	Week 12		Week 0	Week 12	
ADA+EEN	10	2.69 ± 0.45	2.81 ± 0.51	0.1039	1233.90 ± 136.90	1322.00 ± 150.09	0.0032
ADA+PEN	18	2.79 ± 0.28	2.92 ± 0.34	0.0605	1249.00 ± 136.80	1287.17 ± 151.84	0.0099
*t*	–	0.76	0.67	–	0.28	– 0.58	–
*P*	–	0.4542	0.5096	–	0.7818	0.5643	–

*ADA+EEN*: Adalimumab combined with exclusive enteral nutrition, *ADA+PEN*: Adalimumab combined with partial enteral nutrition

**Fig. 2:**
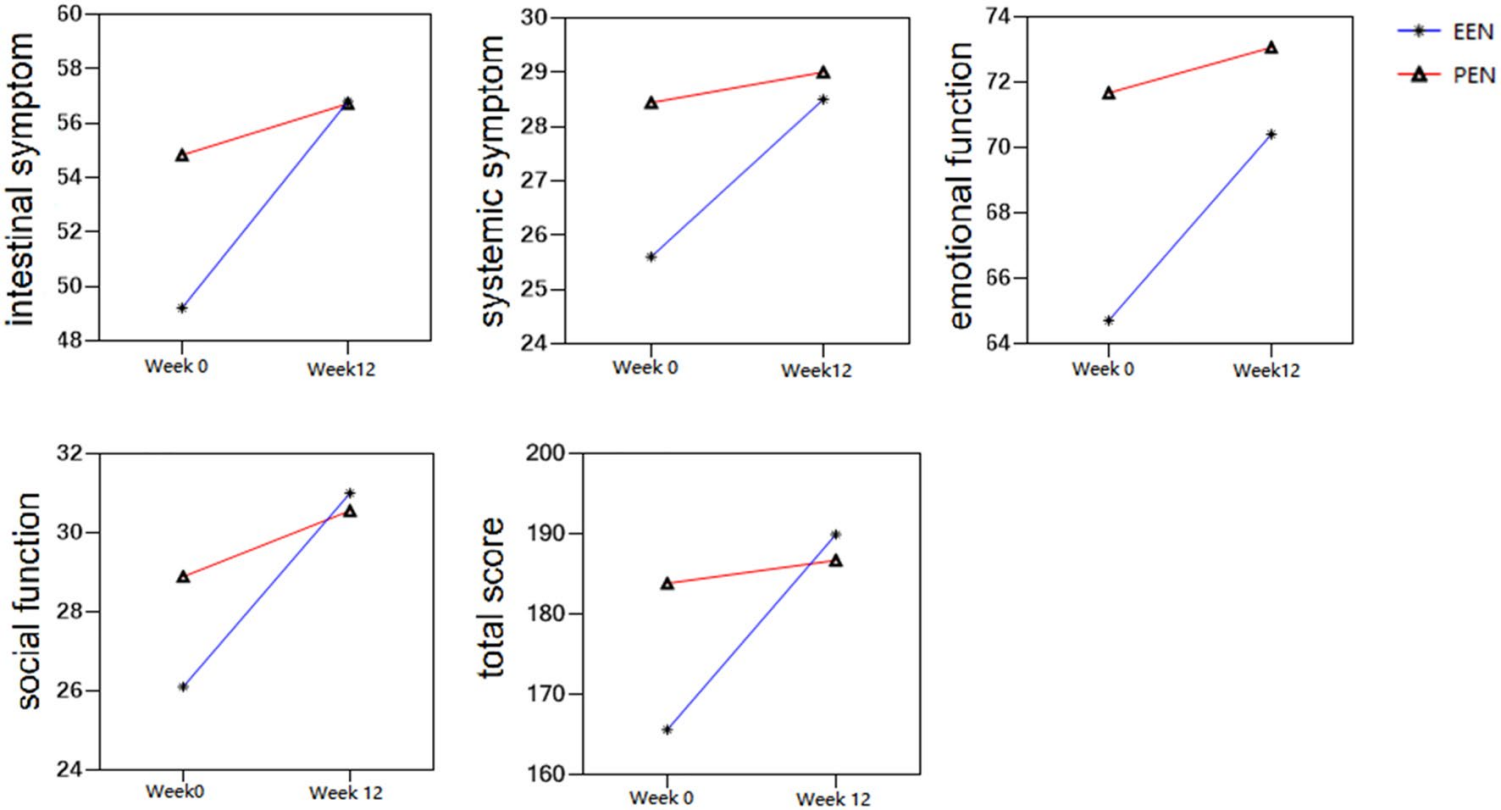
Scores on the intestinal symptoms, systemic symptoms, emotional function and social function dimensions and the total score were compared between the ADA+PEN group and ADA+EEN group at week 0 and week 12

The IBDQ included 32 questions across four dimensions such as intestinal symptoms, systemic symptoms, emotional function and social function. The total score was also obtained. Figure 2 shows that the scores on the four dimensions and the total scores of patients in the ADA+PEN and ADA+EEN groups showed an upward trend during the treatment period. There was no significant difference in scores between the two groups at baseline and at week 12 (*p* > 0.05). There were significant differences in intestinal symptoms, emotional function, social function and total score from W0 to W12 in the ADA+EEN group (*p* < 0.01). At the end of the induction phase, patients in the ADA+PEN group still had higher scores on the emotional function and systemic symptoms dimensions than those in the ADA+EEN group. However, self-ratings of intestinal symptoms were the same in the two groups (Fig. 2).

The EQ-5D-5L consists of mobility, self-care, and usual activities, pain/discomfort, anxiety/depression and the EuroQol visual analog scale (EQ-VAS) was used to assess patients’ health status. After treatment, the scores of 5 aspects in the two groups were lower than those before treatment, and the EQ-VAS score was higher than that before treatment. At baseline, only anxiety/depression was significantly different between the ADA+PEN group and ADA+EEN group but there was no significant differences in other aspects between the two groups after treatment (*p* > 0.05). Anxiety/depression and pain/discomfort in the ADA+EEN group were significantly different before and after treatment from W0 to W12, which were [(2.10 ± 0.88) vs (1.50 ± 0.71)] and [(1.70 ± 0.82) vs (1.30 ± 0.67)], respectively. However, only the EQ-VAS score increased significantly in the ADA+PEN group at week 12[ (90.39 ± 7.90 vs 82.67 ± 16.06)] (*p* < 0.05). There were no statistically significant differences in the five aspects and EQ-VAS scores between ADA+PEN group and ADA+EEN group at week 12 (Table 5).

**Table 5:** Mobility, self-care, usual activities, pain/discomfort, anxiety/depression and EQ-VAS were compared between the ADA+PEN group and ADA+EEN group at week 0 and week 12

Group	*n*	Mobility		*P*	Self-care		*P*	Usual activities		*P*
		Week 0	Week 12		Week 0	Week 12		Week 0	Week 12	
ADA+EEN	10	1.30 ± 0.67	1.30 ± 0.67	1.0000	1.10 ± 0.32	1.10 ± 0.32	1.0000	1.40 ± 0.70	1.30 ± 0.48	0.3434
ADA+PEN	18	1.17 ± 0.38	1.00 ± 0.00	0.0827	1.06 ± 0.24	1.00 ± 0.00	0.3313	1.22 ± 0.55	1.00 ± 0.00	0.1037
*t*	–	− 0.58	− 1.41	–	− 0.42	− 1.00	–	− 0.75	− 1.96	–
*P*	–	0.5755	0.1934	–	0.6757	0.3434	–	0.4628	0.0811	–

Group	*n*	Pain/discomfort		*P*	Anxiety/depression		*P*	EQ-VAS		*P*
		Week 0	Week 12		Week 0	Week 12		Week 0	Week 12	
ADA+EEN	10	1.70 ± 0.82	1.30 ± 0.67	0.0368	2.10 ± 0.88	1.50 ± 0.71	0.0051	73.10 ± 19.96	78.30 ± 21.39	0.1107
ADA+PEN	18	1.61 ± 0.70	1.50 ± 0.51	0.6073	1.28 ± 0.46	1.44 ± 0.51	0.1872	82.67 ± 16.06	90.39 ± 7.90	0.0348
*t*	–	− 0.30	0.88	–	− 2.76	− 0.24	–	1.39	1.72	–
*P*	–	0.7642	0.3860	–	0.0173	0.8121	–	0.1777	0.1145	–

*ADA+EEN*: Adalimumab combined with exclusive enteral nutrition, *ADA+PEN*: Adalimumab combined with partial enteral nutrition

## Discussion

CD is a chronic recurrent disease. It occurs more frequently in young people, and the onset is insidious. During the early stage, it manifests as only intermittent abdominal discomfort or is asymptomatic. CD can occur in any part of the digestive tract but mainly affects the end of the ileum and colon; later, it can later seriously interfere with intestinal functions and cause irreversible damage [12]. During the occurrence and development of CD, a variety of inflammatory factors, including anti-tumor necrosis factor (TNF), play an important role. At present, biological agents that specifically inhibit TNF-α have been widely used in the induction and maintenance treatment of patients with moderate to severe CD after the failure of conventional treatment and are recommended as first-line biological therapy by the American College of Gastroenterology (ACG) guidelines, the European Organization for Crohn’s Disease and Colitis (ECCO) guidelines and other authoritative guidelines worldwide. According to the 2018 new ACG guidelines, anti-TNF agents can be used as initial treatment for severe symptomatic active CD [2, 13–17].

At present, EEN and PEN effectively ameliorate clinical symptoms in pediatric CD patients, but their efficacy in adult CD patients is still unclear [18]. Sigall-Boneh et al. compared the remission effect of PEN in children and adults with CD and showed that after 6 weeks of treatment, the clinical remission rates of children and adults with CD were 70% and 69%, respectively, and the difference was not statistically significant [19]. In this prospective study, 56 patients with CD were divided into the ADA+EN group and the ADA  group. The results showed that both treatments could induce remission of active CD. After treatment, all clinical indexes such as fecal calprotectin, Hb, Plt, ESR, Alb, BMI, CRP, CDAI, SES-CD score and Alb were significantly improved in the ADA+EN group. However, fecal calprotectinand, Hb, SES-CD scores and Alb did not improve significantly in the ADA group.   The fecal calprotectin and CDAI scores were significantly lower in the ADA+EN group than in theADA group at week 12. The level of albumin in patients was also higher in the ADA+EN group than in the ADA group. Crohn’s disease (CD) patients have long-term insufficient intake of calories and various nutrients due to intestinal inflammatory activity, recurrent diarrhea and other factors, and most patients will have varying degrees of malnutrition. By combining biological agents and enteral nutrition therapy, CD patients can reduce the burden on the digestive tract and meet their daily energy requirements faster and more comprehensively. In addition, given the interaction of multiple factors in IBD, comprehensive assessment of inflammation and intestinal barrier destruction should also consider nutritional factors, such as fiber intake, malnutrition and abnormal weight, which play an important role in the pathogenesis of IBD. Therefore, the necessity of nutritional therapy should be emphasized in the overall treatment of CD patients to achieve clinical remission and mucosal healing [20]. The use of specialized enteral nutrition therapy in combination with infliximab appears to be more effective at inducing and maintaining clinical remission among patients with Crohn's disease than infliximab monotherapy [21].Results of the 102 adult CD patients who met the inclusion criteria, 45 were in the EN group (> 900 kcal/day EN) and 57 were in the non-EN group (< 900 kcal/day EN or no EN at all). The cumulative remission rate was significantly higher in the EN group than in the non-EN group (*p* = 0.009) [22].Therefore, biological agents combined with EN are recommended for treatment in the induction period of CD.

Patients diagnosed with active CD were offered EEN for 12 weeks. After 12 weeks of EEN, 80.5% of patients achieved full clinical remission totally and 47% achieved mucosal healing after the treatment [6]. A meta-analysis that included four randomized controlled trials (*n* = 144) in active Crohn’s disease found no significant difference in the remission rates between those treated with enteral nutrition and those treated with corticosteroids (relative risk, RR 0.97, 95% CI 0.7–1.4, random effect model) [23]. EEN is superior to corticosteroids in improving short-term mucosal inflammation and reducing the PCDAI index [24]. Although EEN has a good safety but patient adherence and tolerance are poor, resulting in many patients unable to maintain treatment.One study proposed that both CDED plus PEN and EEN were effective in inducing remission and significantly reducing inflammation. However, CDED + PEN was more well tolerated with sustained remission and reduced inflammation at week 12 [25]. A prospective study compared the efficacy of PEN and 6-mercaptopurine in the maintenance treatment of CD. After 24 months of follow-up, the efficacy of nutrition (providing more than 50% energy) plus mesalazine in maintaining remission was significantly higher than that of mesalazine monotherapy but was equivalent to that of 6-mercaptopurine plus mesalazine [26]. A total of 37 patients in the biological agent combined with EN group were divided into the ADA+EEN group (*N* = 16) and the ADA+PEN group (*N* = 21). The results showed that both treatments could induce remission of active CD, but there was no significant difference in the experimental indexes between the two subgroups at week 12. There was no significant difference in the induction of remission of CD between the biological agents combined with PEN and EEN group.

In a more severely malnourished group of patients with inflammatory bowel disease, Christie and Hill showed that patients had lost (on average) 18.4 kg of body weight, including 35% protein and 32% fat,compared with that of controls [27]. The physical condition of patients in the ADA+EEN group and ADA+PEN group was evaluated although body composition analysis including body fat percentage, muscle, body water, inorganic salts and BMR was performed at week 0 and week 12.  These parameters were significantly higher after treatment in both subgroups.  After 3 weeks of enteral nutrition, nutritionally compromised patients showed significant and proportionate gains in body protein stores in addition to body fat and water. This suggests an important role for enteral nutrition in the repletion of malnourished patients [28]. There was no significant difference in body water, inorganic salts or BMR between two groups at week 12. There was no significant difference in the health status of patients with active stages of CD between the biological agents combined with PEN subgroup and the EEN subgroup.

 The IBDQ questionnaire was developed by Guyatt et al. in 1989 to evaluate the health status of patients with IBD. It includes 32 questions across four dimensions: intestinal symptoms, systemic symptoms, emotional function and social function. Each question is answered on a seven point scale, and the total score ranges from 32 to 224 where higher scores represent better health status [29]. The IBDQ questionnaire provides accurate and comprehensive assessment information for clinical work from the perspective of patients to understand patients’ symptoms, psychological burden, feelings and quality of life. Compared with EEN, PEN is more attractive to patients because the food on the table can also be consumed. This treatment can significantly reduce the psychological burden of patients and improve their quality of and perspective on life. From this prospective study, it can be seen that the ADA+EEN group exhibited improved intestinal symptoms, emotional function and social function to a greater extent than the ADA+PEN group (*p* < 0.01). This may be related to the complete restriction of regular dietary intake during EEN. Similar to partial enteral nutrition, the scores on the four dimensions and the total score of IBDQ showed an upward trend during treatment. The self-rated scores of intestinal symptoms were similar between the two groups at week 12, indicating that PEN did not aggravate intestinal symptoms compared with EEN.

The EQ-5D-5L was used to analyze mobility, self-care, usual activities, pain/discomfort, and anxiety/depression, and the EQ-VAS was used to evaluate the health status of patients. Each dimension contained five levels: no difficulty, a little difficulty, moderate difficulty, severe difficulty and very severe difficulty/unable to perform, respectively. Higher scores on the five dimensions indicate worse health. Table 5 shows that both ADA+PEN group and ADA+EEN group can improve the health status of patients after treatment. Table 5 shows that the ADA+EEN group can reduce pain/discomfort and anxiety/depression to a greater extent after treatment but there was not significant difference between the two groups at the end of the induction period at week 12. Research has demonstrated the ability of EEN which is a highly restrictive and nutrition-based treatment to result in a significant decrease in inflammation and improve quality of life in CD patients. However, the inherent heterogeneity of the foods has brought challenges [30].

Comparisons of BMI, CRP, fecal calprotectin, Hb, Plt, ESR, CDAI scores, SES-CD scores, Alb and drug concentrations indicated that EN combined with biological agents yields a better therapeutic effect than the use of biological agents monotherapy in the induction period. ADA+EEN group and ADA+PEN group can also improve the remission rate of patients with active stages of CD, and there were no significant differences in BMI, CRP, calprotectin, Hb, PLT, ESR, CDAI score, SES-CD score, Alb, drug concentration or body composition between the two groups. According to the IBDQ and EQ-5D-5L questionnaires, PEN therapy can significantly improve the patients’medical adherence, psychological burden and quality of life. These data support the use of ADA+PEN as a first-line therapy during the induction of remission in CD.

Indeed, several limitations to our study exist, the most important one being the small number of patients who completed the study protocol. Patients reported about their own adherence to the prescribed volume of enteral formula and this introduces some reporting bias. Therefore, the results should be interpreted with caution and further larger studies are needed to assess the rates of endoscopic remission and mucosal healing after PEN treatment, before any firm conclusions should be drawn.

## Data Availability

The data underlying this article are available in the article and in its online supplementary material.
